# Latest Advances and Future Challenges in Pancreatic Surgery

**DOI:** 10.3390/jcm12010371

**Published:** 2023-01-03

**Authors:** Gaëtan-Romain Joliat

**Affiliations:** Department of Visceral Surgery, Lausanne University Hospital CHUV, University of Lausanne (UNIL), Rue du Bugnon 46, 1011 Lausanne, Switzerland; gaetan.joliat@gmail.com

The field of pancreatic surgery has considerably evolved in recent decades. The current postoperative mortality rates have drastically improved and presently range between 2% and 8% [1]. The postoperative overall morbidity has also decreased but remains approximately 50–70% [1]. To achieve these results, several advances were undertaken. Perioperative management has become increasingly standardized, particularly due to the development of enhanced recovery after surgery (ERAS) pathways [2]. Technical progress, such as the development of minimally invasive techniques (laparoscopy and robotics), improvements in anesthesia, and applications of evidence-based perioperative care have also contributed to efforts to render pancreatic surgery safer and less morbid. Furthermore, all these advances have permitted us to perform more invasive operations, such as multi-visceral resections and vascular resections, with promising results among well-selected cases.

A major element of the current research in pancreatic surgery concerns the pathophysiology and prediction of the postoperative morbidity. In particular, specific post-pancreatectomy complications, such as delayed gastric emptying, pancreatic fistula, or hemorrhage, are important burdens affecting the patients’ quality of life, length of hospital stay, and overall costs [3]. Even though the pathological mechanisms and risk factors of postoperative complications are now better described and deciphered, improvements aiming to decrease their incidence and to anticipate their occurrence are crucially required. In this regard, specific tailored and individualized perioperative care algorithms and artificial intelligence might help us to reduce the postoperative morbidity rate and the consequences of particular complications.

Regarding minimally invasive surgery, the use of robotics for pancreatectomies has emerged and developed in recent years. It has been shown to offer several advantages for the patient and ergonomic benefits for the surgeon [4]. Moreover, certain studies have shown that the costs involved are not a point of argument in the long term [5]. Hopefully, with the rapid development of robotics, surgical robots will become less expensive and more widely available in the future. Robotic surgery will certainly form part of the armamentarium of pancreatic surgeons in the future. The present challenge lies in the training of young pancreatic surgeons in robotic surgery. Important progress has also been made in the medical oncology of pancreatic cancers. For instance, neoadjuvant treatments for specific patients (e.g., borderline resectable pancreatic cancer or locally advanced pancreatic cancer) have shown interesting and promising results to date [6]. The development and improvement of chemotherapy, targeted therapy, and immunotherapy will synergize pancreatic surgery even more efficiently and certainly help to improve the long-term outcomes of patients with pancreatic cancers.

It is therefore an exciting time to work in the field of pancreatic surgery, in which technology (e.g., robotics, artificial intelligence, indocyanine green fluorescence, or augmented reality) has considerably improved and developed in order to help the surgeon to achieve the best possible outcomes for the patients. The greatest challenges in the near future will be to incorporate all the technological tools so as to tailor the management of the disease to individual patient idiosyncrasies, without omitting the important and necessary human factor.

The following Special Issue, entitled “Surgery for Pancreatic Diseases: Recent Progress and Future Directions”, specifically focuses on the latest advances in, and the evolution of, pancreatic surgery.
